# Surgical techniques in restoration lumbar lordosis: a biomechanical human cadaveric study

**DOI:** 10.1007/s43390-022-00549-x

**Published:** 2022-08-11

**Authors:** A. E. A. Ochtman, A. Bisschop, R. L. A. W. Bleys, F. C. Öner, S. M. van Gaalen

**Affiliations:** 1 Department of Orthopedics, University Medical Center Utrecht, PO box 85500, 3508 GA Utrecht, The Netherlands; 2 Department of Orthopedics, VU University Medical Center, Amsterdam, The Netherlands; 3 Department of Anatomy, University Medical Center Utrecht, Utrecht, The Netherlands; 4 Acibadem International Medical Center, Arlandaweg 100, 1043 HP Amsterdam, The Netherlands

**Keywords:** Lordosis restoration, Sagittal spinal alignment, Biomechanical, Cadaveric

## Abstract

**Purpose:**

Degenerative changes of the lumbar spine lead in general to decrease of lumbar lordosis (LL). This change affects the overall balance of the spine, and when surgery is deemed, necessary restoration of the LL is considered. How this restoration can be achieved is a matter of controversy. The main purpose of this cadaveric study was to investigate the different steps of common posterior surgical techniques to understand the contribution of each successive step in restoring LL.

**Methods:**

Ten fresh-frozen human lumbar spine specimens were used to perform a sequential correction and instrumentation with a pedicle screw construct.

**Results:**

The mean LL angle measured at L3–L4 in intact condition was 12.9°; after screw insertion and compression, this increased to 13.8° (+ 7%, *p* = 0.04), after bilateral facetectomy to 16.3° (+ 20%, *p* = 0.005), after discectomy and insertion of interbody cage to 18.0º (+ 9%, *p* = 0.012), after resection of the lamina and the processes spinosus to 19.8° (+ 10%, *p* = 0.017), and after resection of the anterior longitudinal ligament to 25.4° (+ 22%, *p* = 0.005).

**Conclusions:**

Each step contributed statistically significant to restoration of segmental lordosis with bilateral facetectomy contributing the most in terms of percentage.

**Level of Evidence:**

IV.

## Introduction

Lumbar degenerative disorders, such as degenerative disc disease, degenerative spondylolisthesis, and degenerative scoliosis, can lead to anatomical changes and affect up to 60% of the aging adult population and is the most common cause of disability in patients between 45 and 65 years old [1, 2]. Degeneration of the lumbar spine is characterized by osteophytes formation, reduced disc height, and, in some cases, spinal stenosis. Decrease of lumbar lordosis has been found with increase of age [3–9]. This loss of lumbar lordosis (LL) affects the overall balance and thereby the biomechanics of the whole spine [10]. From L1 to sacrum, the contribution of the lordosis increases with every segment. Janik et al. stated that two-thirds of the total LL are located in the lower two levels (L4-5 and L5-S1) and 85% is found in the L3-S1 segments [11]. The value of LL is highly variable in the general population and becomes even wider with increasing age, which may explain why some patients stay relatively asymptomatic, while others complain about significant functional disability and pain [12].

Optimal treatment remains controversial [13, 14]. One of the most frequent indications for surgical management is neurologic symptoms. More relative indications are severe disability despite conservative treatment such as physical therapy and neurogenic claudication. A multicenter randomized controlled trial by Fritzell et al. showed better clinical outcome for spinal fusion over non-surgical treatment [15]. However, comparative evidence demonstrating superiority of one spinal fusion technique over another is lacking [16]. Posterolateral fusion (PLF) has been considered as golden standard surgical treatment for many years. [17] Although, with the increasing attention for sagittal alignment of the spine over the last decades, lumbar interbody fusion (LIF) has increased in popularity due to the theoretical advantage of restoring the disc height and thus the LL [18]. Lumbar fusion in hypolordosis or even kyphosis is widely associated with adjacent segment degeneration. According to several cadaveric studies, insufficient restoration of lordosis leads to degenerative changes in the adjacent segments [19–21] which has been confirmed in clinical studies as well [2, 22, 23]. Lazennec et al. showed post-fusion persistent pain to be significantly related to insufficiently restored LL, independent of other factors such as non-union [24]. Therefore, restoring the LL is considered to be one of the main goals of spinal fusion to improve clinical outcome.

Most studies comparing clinical outcomes of different surgical techniques focus on fusion rate rather than adequate lordosis restoration [25]. To quantify what surgical technique is most appropriate to restore lordosis, we investigated the different steps of posterior approach in an experimental setup to understand the contribution of each successive step in restoring LL.

## Materials and methods

### Specimens and specimen preparation

Twenty-one freshly frozen (− 20°) human cadavers (mean age: 792 years, range: 54–89) were screened for testing. The bodies were donated by last will in accordance with the national legislation. Body handling was done according to the guidelines of the Department of Anatomy of the University Medical Center Utrecht.

The specimens were evaluated with conventional radiograms of the lumbar spine. Eleven (52%) specimens with bridging osteophytes, collapsed intervertebral disc spaces, or compression fractures were excluded, which resulted in ten specimens to be used for this study. The specimens were thawed 24 h before testing and lumbar spinal segments (L1-L5) were harvested. Surrounding muscle tissue was carefully removed, keeping the anterior longitudinal ligament as well as the facet joints and interspinous ligaments intact. The cranial and caudal vertebrae were potted in a casting-mold and partially buried in a low melting point (48 °C) bismuth alloy (Cerrolow-147; 48.0% bismuth, 25.6% lead, 12.0% tin, 9.6% cadmium, and 4.0% indium). Adding screws into the vertebral body of the L1 and L5 vertebrae secured fixation into the alloy. All articulating parts were kept free. A three-dimensional system of coordinates was placed on the anterior side of the corpus of L2 to ensure a pure lateral radiogram.

### Testing procedure

**Fig. 1:**
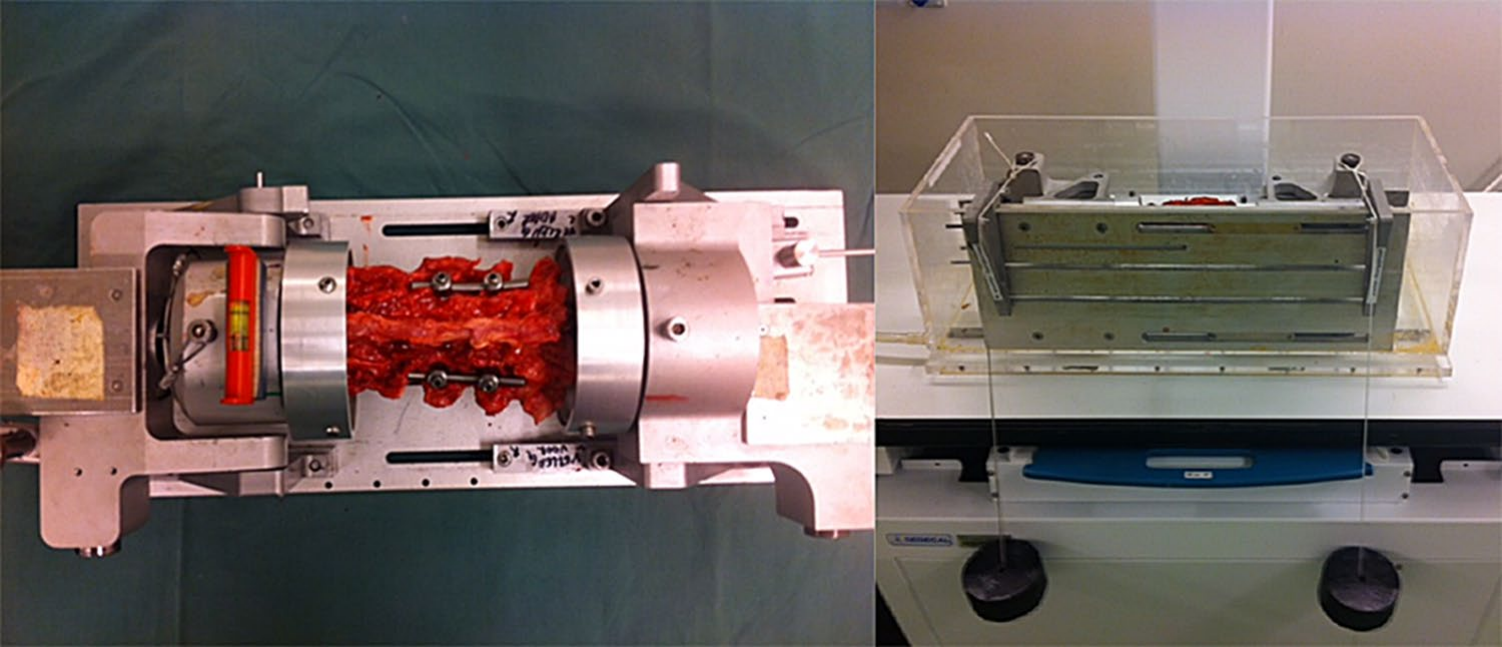
The experimental setup is shown from above (left) and side (right) with lumbar spinal specimen (L1–L5) positioned in the four-point bending device and preloading weights

The test setup was described and validated previously [26, 27]. Lumbar spines were placed horizontally in a custom made 4-point bending device in which pure moments in flexion can be applied (Fig. 1). Loads of 8 kg (785 N) were applied. After 1 min of preloading, a radiogram was made. This setup obtains a physiological condition and guarantees that forces generate a moment that is equal at all levels of the lumbar spine. Throughout the experiment, the spinal specimens were kept moist with 0.9% saline. All tests were performed at room temperature.

At the start of the testing procedure, four pedicle screws were placed at level L3–L4. All steps were performed at this level. The sequence of successive steps was as follows:

1. Screw insertion and connection with roads
2. Bilateral facetectomy
3. Discectomy and cage insertion
4. Complete laminectomy and resection of spinous processes and interspinous ligaments
5. Resection of the anterior longitudinal ligament (ALL)

**Fig. 2:**
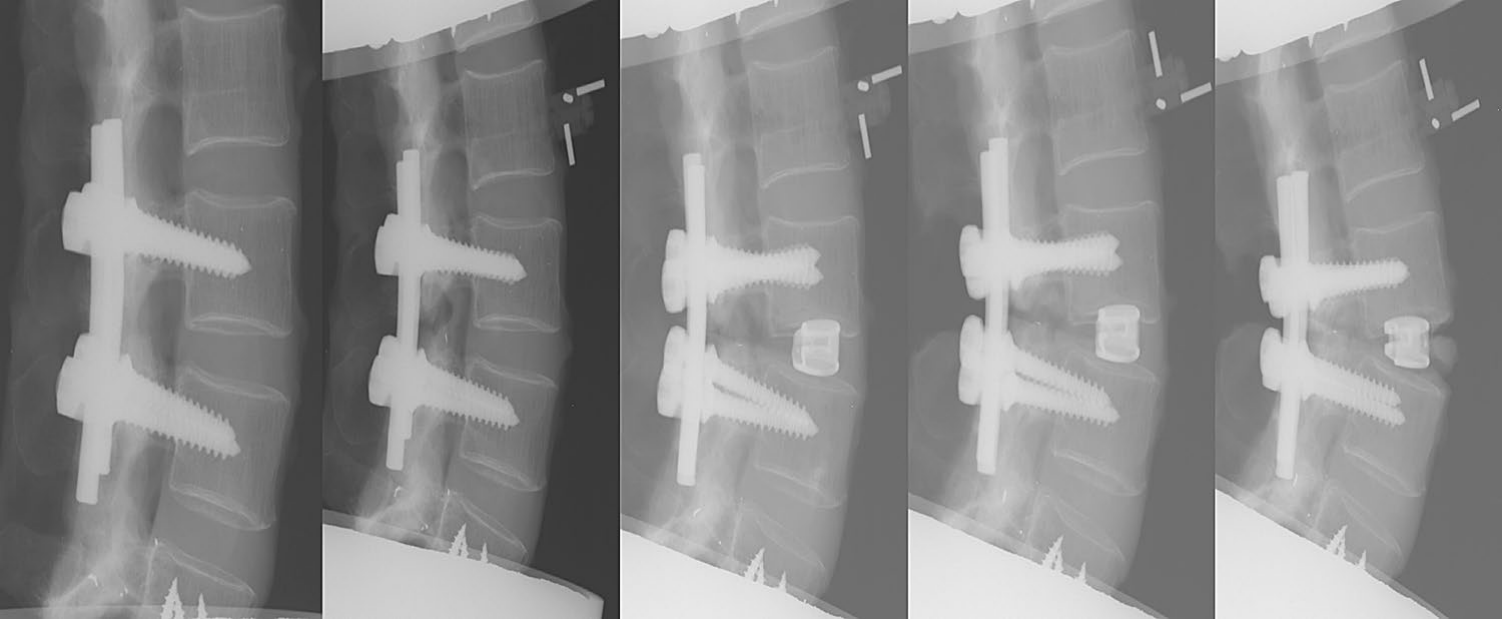
Lateral radiograms of successive steps of testing procedure: from left to right: screw insertion, bilateral facetectomy, cage insertion, laminectomy, and resection of ALL

Compression was given over the pedicle screws at the beginning and after each step by the same researcher. Pure lateral radiograms were obtained before testing and after each step (Fig. 2).

### Data analysis

Radiograms were uploaded in the Picture Archiving and Communication System (PACS). The Cobbs angle was measured between the superior endplate of L3 and the inferior endplate of L4. Measurements were performed three times on consecutive days by the first author. Measurements were used to calculate the mean absolute difference.

### Statistical analysis

Statistical analyses were performed with Statistical Package for the Social Sciences software (SPSS 23.0, SPSS Inc., Chicago, IL, USA). First, data were tested for distribution by the Shapiro–Wilk test. Since data were not normally distributed, analysis was performed using the Wilcoxon signed-rank test. Intra-rater reliability was assessed using intra-class correlation (ICC) coefficients. Statistical significance was set at *p* < 0.05.

## Results

Each successive step resulted in a significant increase of the LL angle. The mean absolute measurements after each step are presented in Table 1. ICC coefficients revealed an excellent intra-rater reliability (ICC = 0.91, *p* = 0.001).

**Table 1:** Absolute measurements of Cobbs angle at L3–L4

	Intact	Screw insertion	Bilateral facetectomy	Disectomy and cage insertion	Laminectomy	Resection of ALL
Mean (°)	12.9	13.8	16.3	18.0	19.8	25.4
SD	3.5	3.9	4.5	5.0	4.6	5.1
Range (°)	7.1–16.9	7.2–21.0	10.0–25.3	10.8–28.8	11.2–28.8	13.9–27.0
Cumulative ∆ (°)		0.9	3.4	5.1	6.9	12.5
%		7%	20%	9%	10%	22%
*p* Value		0.04	0.005	0.012	0.017	0.005

### Angle measurements

**Fig. 3:**
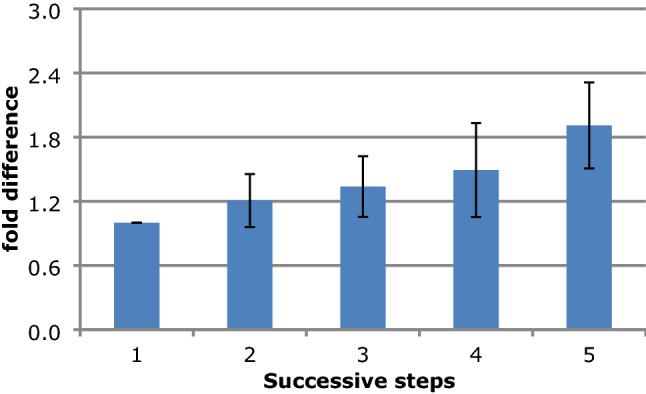
Fold difference analysis

Mean LL angle in intact condition was 12.9°. After screw insertion and compression, the mean angle increased to 13.8° (+ 7%). Bilateral facetectomy resulted in a 3.4° (+ 20%) increase (*p* = 0.005). Discectomy and cage insertion resulted in a further increase of 1.7º (+ 9%) (*p* = 0.012) compared to facetectomy and a total increase of 25% compared with the intact condition. After resection of lamina and processes spinosus, mean LL angle increased to 19.8° (+1.8°, 10%, *p* = 0.017) (+ 30% as compared with intact). The last step, resection of the ALL, resulted in the highest additional increase of 5.6° (+22%) (*p* = 0.005) compared to the previous step. The total increase from the intact condition was 12.5° (+48%). Fold difference analysis is showed in Fig. 3.

## Discussion

Surgical approach for lumbar fusion is an important determinant of achieving lordosis restoration. The traditional Posterior Lumbar Interbody Fusion (PLIF) was first described by Cloward in 1940 and enables a three-column fixation with 360° fusion and anterior support via midline incision [28]. This approach includes a complete laminectomy to visualize and decompress nerve roots in case of neurological symptoms, but facet joints may only be undercut and not further destabilized. In 1982, Harms and Rolinger described the transforaminal lumbar inter body fusion (TLIF) [29]. Neural injury and dural retraction is minimized by the lateral entry point. Originally, a unilateral facetectomy was performed during this surgical technique to insert the cage in the intervertebral disc. However, some spine surgeons remove the facet joints bilaterally [30, 31]. In our study, we found a statistically significant increase in lordosis restoration after bilateral facetectomy. However, we did not compare unilateral with bilateral facetectomy. It has been reported by Tye et al. that there was no significant difference in segmental lordosis between unilateral and bilateral facetectomy [32]. Surprisingly, although no radiographic difference was found, only clinical outcome measurements in the bilateral cohort reached minimally clinical important difference (MCID), which was significantly greater than in the unilateral cohort. Other factors contributing to the improvement of clinical outcome in bilateral resection of facet joints to explain this improvement could be reducing radicular pain by a complete foramina decompression or the phenomenon that the facet joints themselves are the cause of the pain [33]. More recent, Snynder et al. found a statistically significant improvement of lordosis angle after complete bilateral facetectomy compared with unilateral facetectomy in seven cadaveric specimen, although this difference might not be clinically relevant as it was only 1.06° [34]. No previous studies have been found in the literature to compare our results of laminectomy contributing to the restoration of LL, as this is mainly performed to decompress nerve roots in patients with neurological deficit. Although, laminectomy alone (without posterior fixation) is associated with a decrease of total LL during long-term follow-up and the rate of reoperation is higher compared with laminectomy with fixation [35].

Our results showed a surprisingly small contribution of discectomy and cage insertion (+9%), although it was still statistically significant. We used the lordotic PLIF cage that best fitted the intervertebral space (11° in 2 specimens, 13° in 8 specimens) and placed it in the anterior third to the best as possible, so we hypothesized a greater contribution. This difference could be partly explained by insufficient discectomy which led to cage placement relatively posterior in three specimens. Therefore, we performed a subanalysis without these three specimens and found a mean increase of 4.8° (3.1° more than in the analysis with all 10 specimens, *p* = 0.001) compared with bilateral facetectomy only. In this analysis, laminectomy and cage insertion contributes significantly more to the total lordosis restoration (21%). These results underline not only the clinical relevance of introducing a cage to restore LL but also the importance of placement in the anterior third of the intervertebral space. Furthermore, correct placement of a cage offers a biomechanical advantage, as well: it is subject to a compressive load since the anterior column supports most of the body load. Combined with either allograft or autogenous bone graft densely impacted within or next to the cage, bony fusion is stimulated.

This study showed a statistically significant increase of segmental lordosis at level L3–L4 after each step, with a total increase of 12.5° (49%) compared to the intact condition. The biggest contribution was found with resection of the ALL (+22%). This is consistent with prior studies, although the increase in our study was less. Uribe et al. demonstrated in a cadaveric study that sectioning the ALL and use of a lordotic cage can provide increase in segmental lordosis roughly equivalent to a Smith–Peterson osteotomy (up to 13.1° in a normal cadaveric spine) [36]. The ALL is normally only sectioned during an anterior lumbar interbody fusion (ALIF) and is said to be most effective in restoration of LL [37]. However, the anterior approach is associated with concerning complications such as retrograde ejaculation in male, ureter injury, and major vessel injury to the blood or lymphatic circulation [37, 38] and is mostly performed at levels L4-L5 and L5-S1. Although, more recent literature shows good clinical results for lumbar fusion from L1 to S1 [39]. Our results regarding resection of the ALL should be interpreted with care when comparing to results of ALIF in the literature as this was the last step after several posterior releases that have biased the outcome. Nevertheless, the increase of 5.7° after these posterior releases did show that the ALL was the restricting factor for a further increase in segmental lordosis restoration after bilateral facetectomy, cage insertion, and laminectomy. This suggests that an ALL release from posterior could be an important last step of a PLIF procedure to restore the maximum amount of LL.

One of the limitations of our study is that we performed the different steps in the same order for each specimen and could therefore not correct for order effects. This may have overrated the effect of the last step but let to statistically significant results with a relatively small cohort. Another limitation is that we specifically selected specimen without any significant signs of degeneration to avoid biased results due to stiffness and facet joint hypertrophy. In the aging spine, with disc degeneration and end plate changes, the results might be different.

In conclusion, the results presented here show increase in segmental lordosis after each step performed during an instrumented PLIF procedure. Bilateral facetectomy was found to contribute the most in terms of percentage to restoration of LL of the posterior steps.
